# Emerging *Enterococcus* isolates in postoperative endophthalmitis by selection pressure of fluoroquinolones: an 11-year multicenter and experimental study

**DOI:** 10.1080/22221751.2020.1810134

**Published:** 2020-08-28

**Authors:** Jiyeun Kate Kim, Ki Yup Nam, In Young Chung, Woo Jin Jeung, Yoon Hyung Kwon, Jung Min Park, Yong Seop Han, Ji Eun Lee, Ik Soo Byon, Sung Hu Park, Hyun Wong Kim, Kang Yun Park, Hee Sung Yoon, Indal Park, Han Woo Kim, Sang Joon Lee

**Affiliations:** a Department of Microbiology, College of Medicine, Kosin University, Busan, South Korea; b Department of Ophthalmology, Chungnam National University, Sejong Hospital, Sejong, South Korea; c Department of Ophthalmology, College of Medicine, Gyeongsang National University, Jinju, South Korea; d Department of Ophthalmology, College of Medicine, Dong-A University, Busan, South Korea; e Department of Ophthalmology, Maryknoll Hospital, Busan, South Korea; f Department of Ophthalmology, Graduate School of Medicine, Busan National University, Busan, South Korea; g Department of Ophthalmology, College of Medicine, Inje University, Busan, South Korea; h Sungmo Eye Hospital, Busan, South Korea; i Department of Ophthalmology, College of Medicine, Kosin University, Busan, South Korea

**Keywords:** Selection pressure, *Enterococcus*, fluoroquinolone, antibiotic resistance, endophthalmitis

## Abstract

Postoperative endophthalmitis (PE) is the devastating complication that frequently results in vision loss. Recently, *enterococcus* have emerged as a major cause of PE in several countries and resulted in poor visual outcome. However, the reason remains elusive. We investigate whether selection pressure of fluoroquinolone exerts effects on microorganism profiles isolated from PE. Medical records of patients who were diagnosed with PE at eight resident training institutions between January 2004 and December 2015 were reviewed. The most common isolate was *Enterococcus faecalis* (28.0%), followed by *Staphylococcus epidermidis* (18.6%) and other coagulase negative *Staphylococci* (7.6%). However, the rates of *E. faecalis* isolated from conjunctival microbes were 6.2% (16/257) and their resistance to fluoroquinolones was higher than those of *S. epidermidis*. *In vitro* and *in vivo* co-culture models of *E. faecalis* and *S. epidermidis* were established for survival assays after administration of fourth-generation fluoroquinolone. In *in vitro* co-culture model, the survival assay of *E. faecalis* and *S. epidermidis* against the treatment of moxifloxacin showed that *E. faecalis* survived significantly better than *S. epidermidis* in the presence of moxifloxacin 1 µg/mL and more. In *in vivo* co-culture model, *E. faecalis* survived significantly better than *S. epidermidis* after topical treatment of moxifloxacin (5 mg/mL). *E. faecalis* has been the most common causative strain of PE in Korea. We suggest that the increase of *E. faecalis* in PE could be associated with the selection pressure of fourth-generation fluoroquinolone.

**Summary:** *Enterococcus* spp. have emerged as a leading causative strain of postoperative endophthalmitis in 11-year clinical data. We suggest that the increase of *Enterococcus* spp. is associated with the selection pressure of fourth-generation fluoroquinolone.

## Introduction

Postoperative infectious endophthalmitis (PE) is the most devastating complication after intraocular surgery. Cataract surgery is the most common cause of PE. In a recent large meta-analysis, the incidence of PE ranged from 0.012% to 1.3% [1,2]. Infectious endophthalmitis is initiated by the entrance of microorganisms into the intraocular space, and the type of microorganism is a critical factor in determining visual outcome [3,4].

So far, the most frequent causative strain of PE has been known as coagulase negative *staphylococci* (CNS), including *Staphylococcus epidermidis* [5,6]. Recently, *Enterococcus* spp. have emerged as a major causative strain of PE in Sweden, Korea, and Taiwan [4,7,8]. The reason underlying the increase of *Enterococcus* spp. has not been fully understood, but several studies speculated that an important contributory factor might be the selection pressure from increased consumption of broad-spectrum antibiotics perioperatively [4,9,10]. *Enterococcus* spp. are of particular clinical importance because they cause a fulminant and destructive disease course and poor visual outcomes [11–13]. Therefore, the emergence of *Enterococcus* spp. as a major cause of PE would negatively impact the expectations for visual outcomes.

*Enterococcus* spp. have an intrinsic resistance to multiple antibiotics such as cephalosporins, aminoglycosides, and fluoroquinolones [14–16]. For the broad-spectrum topical antibiotics used to prevent PE, fourth generation fluoroquinolones are commonly used perioperatively without clear evidence [17,18]. Fluoroquinolones show poor or moderate antimicrobial activity against *Enterococcus* spp. [14,19].

Despite the increasing importance of *Enterococcus* spp. in PE, clinical data about the susceptibility of *Enterococcus* spp. isolated from eye to various fluoroquinolones is scarce. The Clinical and Laboratory Standard Institute (CLSI) does not provide a resistance cut-off value of all generation fluoroquinolones for *Enterococcus* spp*.*, and the published studies on the antibiotics susceptibility of *Enterococcus* spp. to fluoroquinolone used various methods depending on each hospital with different cut-off values [20]. Moreover, there has been no clear investigation to address whether the domination of *Enterococcus* spp. of PE was caused by their intrinsic resistance toward fluoroquinolone.

In this study, the causative isolate of PE was investigated using 11-year clinical data, and their antibiotic susceptibility was analysed. Two main causative bacteria, *Enterococcus faecalis* and *S. epidermidis*, were isolated from conjunctival microbes and tested for antibiotics susceptibility on fluoroquinolones*. In vitro* and *in vivo* co-culture models of *E. faecalis* and *S. epidermidis* were established and used for survival assay to investigate whether the selection pressure of fourth-generation fluoroquinolone has effects on the domination of *E. faecalis*.

## Materials and methods

### Clinical data

We reviewed medical records of patients with PE after operation for cataract, glaucoma, or vitreous in seven resident training institutions in Korea from January 2004 to December 2015. Institutional review board (IRB) approval was obtained from Gyeongsang National University, and the protocol of this study adhered to the provisions of the Declaration of Helsinki. Diagnosis was based on the doctor’s discrimination by the clinical manifestations of patients, and microbiologic cultures were performed by aspiration of the aqueous humour or vitreous. For the clinical analysis of PE, the following data were evaluated: case numbers of presumptive and culture-positive endophthalmitis, operation, and isolated microorganisms and their antibiotic susceptibility. Culture and identification method was described in detail in the supplementary data.

### Collection of conjunctival microbes

Specimens were obtained from 208 patients (416 eyes) who were scheduled for cataract surgery or intravitreal injection at the Gospel Hospital, Kosin University from April 2014 to January 2018. All contact lens wearers, patients with any inflammatory or infectious ocular surface, or any recent (at least 3 months) users of antibiotics that could alter the status of conjunctival flora were excluded. The patient was asked to look up, and the inferior cul-de sac was swabbed using a sterile cotton tip without touching the eyelid margin or lashes. The specimens were inoculated directly in 5% blood agar plates at the bedside and incubated at 37°C for 3∼7 days. Each colony was collected and cultured on the different culture dishes. Cultured bacteria were identified in the same manner. All isolated strains of *E. faecalis* and *S. epidermidis* were further examined for antibiotic susceptibility by broth micro-dilution method which was described in detail in the supplementary method.

### Survival assay in *in vitro* co-culture model

The co-culture bacterial solution of standard strains of *S. epidermidis* and *E. faecalis* were prepared to have equal concentration of both bacteria (2.5 × 10^7 ^CFU/mL) in TSB media and incubated at 37°C for 14^ ^h. Then, the 100 µL of co-cultured solution was diluted with 900 µL of TSB media containing various concentrations of moxifloxacin (0∼16 µg/mL, Tokyo Chemical Industry, Japan) and further incubated for 6^ ^h. After incubation with moxifloxacin, the bacterial solution was serially diluted and plated on the CDA plate (MB-C1611, MB-cell, Korea), which can differentiate the colonies of *E. faecalis* and *S. epidermidis* by the colours of the colonies; the *E. faecalis* colonies were blue and *S. epidermidis* colonies were white. Bacterial strain and growth conditions were described in detail in the supplementary method.

### Survival assay in *in vivo* co-culture model

Rabbits (New Zealand white rabbit, 4∼5 kg, Hyochang Science, Korea) were used for the *in vivo* co-culture model of *S. epidermidis* and *E. faecalis*. The animals were divided into three groups: the normal control group (NC), co-culture group (CO), and moxifloxacin-treated co-culture group (MC). Each group consisted of five rabbits and only the right eyes were used for experiment. Neither microorganism nor moxifloxacin was administered to the NC group. For the CO and MC groups, rabbits were anaesthetized by intramuscular injection of by tiletamine-zolazepam (Virbac Korea, France) and xylazine (Bayer, Germany), and 9 µL of *S. epidermidis* solution (4X10^9^ CFU/mL) and 1 µL of *E. faecalis* solution (4X10^9^ CFU/mL) were administered into the lower conjunctival sac. To avoid the loss of bacterial solutions, tarsorrhapy was performed using continuous 6–0 nylon (Ethicon, USA). In the MC group, 5 µL of moxifloxacin (0.5%, Tokyo Chemical Industry Co., Japan) was administered to the lower conjunctival sac in similar manner twice at 0 and 1^ ^h after the administration of solutions. In order to compare the number of bacteria among the groups, a sterile cotton swap tip was used to collect the bacteria in the inferior cul-de-sac at 4^ ^h after the administration of bacterial solution. The swapped cotton tips were immediately soaked in 1 mL normal saline in an Eppendorf tube and shaken at 2800 rpm for 60^ ^s using a microtube homogenizer (BeadBug, Benchmark Scientific, USA) to release bacterial cells from cotton tips. Next, the solution was serially diluted and plated on CDA plates for differential colony counting. After overnight incubation at 37°C, the number of colonies of *E. faecalis* and *S. epidermidis* were counted as described above. The study was approved by the IRB and Institutional Animal Care and Use Committee of Kosin University College of Medicine (KMAP-18-19). All experiments that involved animal subjects were performed in accordance with the guidelines and regulations of the Association for Research in Vision and Ophthalmology.

### Statistics

All results were obtained from three independent experiments. One-way ANOVA was used to estimate differences among groups. All experiments were repeated five times to improve reliability. All data are presented as mean ± standard deviation. All statistical analyses were performed with GraphPad Prism (Version 6.03). Statistical significance was defined as *p* < .05.

## Results

### Profile of microorganisms isolated in PE (Table 1)

Two hundred and fifty-three patients of PE were included. The PE was caused by the following surgeries: post-cataract surgery (89.3%, 226 cases), glaucoma operation (4.3%, 11 cases), and vitrectomy (6.3%, 16 cases). The proportion of cataract surgery was highest among the causes of PE. The culture of intraocular specimen was performed in 226 eyes from the total 253 eyes, and the culture positivity rate was 52.2% (118/226 eyes). The most common causative bacterial species was *Enterococcus* spp. (28.0%, 33 cases), followed by *S. epidermidis* (18.6%, 22 cases) and other CNS (7.6%, 8 cases) (Table 1).

**Table 1.** Profile of causative microorganisms cultured from postoperative endophthalmitis.

Isolates of postoperative endophthalmitis					Jan 2004–Jul 2010		Aug 2010–Dec 2015		Jan 2004–Dec 2015	
					*n*	(%)	*n*	(%)	*n*	(%)
Gram (+) cocci					50	(74.6)	37	(72.5)	87	(73.7)
	*Staphylococcus* spp.				22	(32.8)	13	(25.5)	35	(29.7)
		*S. aureus*			2	(03.0)			2	(01.7)
		*S. epidermidis*			14	(20.9)	8	(15.7)	22	(18.6)
		Other CNS			6	(09.0)	3	(05.9)	9	(07.6)
	*Streptococcus* spp.				8	(11.9)	9	(17.6)	17	(14.4)
		*S. pneumoniae*			4	(06.0)	3	(05.9)	7	(05.9)
		Viridans group streptococci			3	(04.5)	6	(11.8)	9	(07.6)
	*Enterococcus* spp.				19	(28.4)	14	(27.5)	33	(28.0)
Gram (+) rods					3	(04.5)	2	(03.9)	5	(04.2)
Gram (-) rods											12	(17.9)	12	(23.5)	24	(20.3)
Fungi					2	(03.0)			2	(01.7)						
	Yeast spp.				1	(01.5)			1	(00.8)						
	*Aspergillus* spp.				1	(01.5)			1	(00.8)						
Total					67		51		118							

Note: Spp., species.

### Antibiotic susceptibility test of microorganisms isolated from PE

The second and third-generation fluoroquinolones showed relatively poor antimicrobial activity against *E. faecalis*, *S. epidermidis*, and other CNS, showing their susceptibility in the range of 42-67% of isolates (Table 2). However, the fourth-generation fluoroquinolone, moxifloxacin, showed excellent activity on *S. epidermidis* and other CNS (Table 2). Unfortunately, moxifloxacin was not included in the antibiotic susceptibility report for *E. faecalis* using automated diagnostics since moxifloxacin was not normally considered to treat *E. faecalis*. *S. epidermidis* and CNS isolates showed no resistance to moxifloxacin, but 57.1% and 54.5% were resistant to ciprofloxacin, 38.5% and 33.3% to ciprofloxacin, and 69.0% and 54.5% to oxacillin, respectively (Table 2).

**Table 2.** Antibiotic susceptibility results of *Enterococcus faecalis*, *Staphylococcus epidermidis*, and other coagulase-negative *staphylococci* (CNS) cultured from postoperative endophthalmitis samples.

Antibiotics	*E. faecalis*			*S. epidermidis*			Other CNS		
	*N*^a^	Susceptibility n^b^ (%)		*N*	Susceptibility n (%)		*N*	Susceptibility n (%)	
TMP-SMX	17	8	(47.1)	27	25	(92.6)	10	9	(90.0)
Ampicillin	26	22	(84.6)	–	–	–	–	–	
Chloramphenicol	17	11	(64.7)	3	3	(100.0)	2	2	(100.0)
Moxifloxacin	10	nd		6	6	(100.0)	1	1	(100.0)
Levofloxacin	21	14	(66.7)	13	8	(61.5)	3	2	(66.7)
Ciprofloxacin	22	13	(59.1)	28	12	(42.9)	11	5	(45.5)
Teicoplanin	20	20	(100.0)	12	10	(83.3)	10	9	(90.0)
Oxacillin	nd	nd		29	9	(31.0)	11	5	(45.5)
Gentamicin	3	0	(00.0)	22	11	(50.0)	11	6	(54.5)
Gentamicin high level	12	4	(33.3)	–	–	–	–	–	
Tobramycin	nd	nd		10	6	(60.0)	–	–	
Vancomycin	22	22	(100.0)	21	21	(100.0)	10	9	(90.0)

Note: TMP-SMX, trimethoprim/sulfamethoxazole; nd, not determined.

**^a^**number of bacterial isolates tested.

**^b^**number of susceptible isolates.

### Microorganisms of conjunctival microbes

To see if there are changes in the normal conjunctival microbes, 416 conjunctiva samples were cultured and 342 microorganisms were isolated. Among isolated bacteria, 257 isolates were Gram-positive bacteria (75.1%), and 85 isolates were Gram-negative bacteria (24.9%) (Table 3). The most common isolate of Gram-positive microorganism was *S. epidermidis* (33.0%, 113 isolates), followed by *Corynebacterium* spp., other CNS, and *E. faecalis*. The number of *E. faecalis* isolates was 16, that is 4.7% of total isolated microorganisms.

**Table 3.** Profile of conjunctival microbes. Matrix-Assisted Laser Desorption Ionization-Time of Flight Mass Spectrometry (MALDI-TOF MS, Bruker Daltonics GmbH, Germany) was used for identification of microorganisms by identification card (VITEK 2 ID Card, bioMérieux, USA).

Isolates of conjunctival microbes (*n*^a^ = 342)					
Gram (+) bacteria	*n*	(%)	Gram (−) bacteria	*n*	(%)
*Staphylococcus epidermidis*	113	(44.0)	*Ochrobactrum* spp.	34	(40.0)
*Corynebacterium* spp.	62	(24.1)	*Pseudomonas* spp.	11	(12.9)
Other CNS	31	(12.1)	*Achromobacter* spp.	8	(09.4)
*Enterococcus faecalis*	16	(06.2)	*Brevundimonas* spp.	7	(08.2)
*Staphylococcus aureus*	15	(05.8)	*Enterobacter aerogenes*	4	(04.7)
*Micrococcus luteus*	6	(02.3)	*Roseomonas gilardii*	4	(04.7)
*Propionibacterium avidum*	4	(01.6)	*Bordetella hinzii*	3	(03.5)
*Kocuria* spp.	4	(01.6)	*Acinetobacter baylyi*	2	(02.4)
*Lactobacillus* spp.	2	(00.8)	*Delftia acidovorans*	2	(02.4)
*Bacillus pumilus*	1	(00.4)	*Proteus mirabilis*	2	(02.4)
*Clostridium bifermentans*	1	(00.4)	*Sphingomonas paucimobilis*	3	(03.5)
*Dermabacter hominis*	1	(00.4)	*Stenotrophomonas maltophilia*	2	(02.4)
*Arthrobacter nicotinovorans*	1	(00.4)	*Cupriavidus pauculus*	1	(01.2)
			*Moraxella* spp.	1	(01.2)
			*Morganella morganii*	1	(01.2)
Total	257		Total	85	

Note: CNS, coagulase negative staphylococci; spp., species.

^a^number of bacterial isolates.

### Antibiotic susceptibility test of *E. faecalis* and *S. epidermidis* isolated from conjunctival microbes

Standard strains of *E. faecalis* and *S. epidermidis* were tested for the antibiotic susceptibility to be used as references for resistance cut-off values (Supplementary Table 1). Our MIC data of *S. epidermidis* and *E. faecalis* match with CLSI data [20]. The CLSI standard cut-off values of antibiotics resistance were used for *S. epidermidis* and *E. faecalis* clinical isolates except for the moxifloxacin resistance cut-off value for *E. faecalis* due to the lack of the CLSI standard data [20]. In our study, we conservatively decided the resistance cut-off value as 2 μg/mL moxifloxacin for *E. faecalis* because it was four times the highest MIC value of *E. faecalis* reference strain.

From the isolates of human conjunctiva, 82 isolates of *S. epidermidis* and 12 isolates of *E. faecalis* were available for antibiotic susceptibility test. The proportion of resistant *S. epidermidis* isolates to ciprofloxacin, levofloxacin, and moxifloxacin was 23.2%, 24.4%, and 17.1% and that of resistant *E. faecalis* was 33.3%, 33.3%, and 33.3%, respectively (Table 4). The proportion of resistant *E. faecalis* to moxifloxacin was approximately two times higher than that of *S. epidermidis*. All breakpoints used here were based on systemic breakpoints provided by CLSI or derived from the method suggested by CLSI. However, the breakpoint for topical therapy has not been established so far, so we conducted survival tests of *S. epidermidis* and *E. faecalis* in vitro and in vivo co-culture model.

**Table 4.** Antibiotic susceptibility test of *S. epidermidis* and *E. faecalis* isolated from human normal conjunctiva to ciprofloxacin, levofloxacin, and moxifloxacin.

		Ciprofloxacin		Levofloxacin		Moxifloxacin	
		*n*^g^	%	*n*	%	*n*	%
*S. epidermidis*	Susceptible^a^	55	(67.1)	55	(67.1)	61	(74.4)
	Intermediate^b^	8	(09.8)	7	(08.5)	7	(08.5)
	Resistant^c^	19	(23.2)	20	(24.4)	14	(17.1)
	Total	82					
*E. faecalis*	Susceptible^d^	8	(66.7)	8	(66.7)	8	(66.7)
	Intermediate^e^	0	(00.0)	0	(00.0)	0	(00.0)
	Resistant^f^	4	(33.3)	4	(33.3)	4	(33.3)
	Total	12					

^a^ciprofloxacin, MIC (μg/mL) ≤1; levofloxacin, ≤1; moxifloxacin, ≤0.5.

^b^ciprofloxacin, 2; levofloxacin, 2; moxifloxacin, 1.

^c^ciprofloxacin, ≥4; levofloxacin, ≥4; moxifloxacin, ≥2.

^d^ciprofloxacin, ≤1; levofloxacin ≤2; moxifloxacin, ≤0.5.

^e^ciprofloxacin, 2; levofloxacin, 4; moxifloxacin, 1.

^f^ciprofloxacin, ≥4; levofloxacin, ≥8; moxifloxacin, ≥2.

^g^number of bacterial isolates tested.

### Survival assay in *in vitro* co-culture model

**Figure 1.**
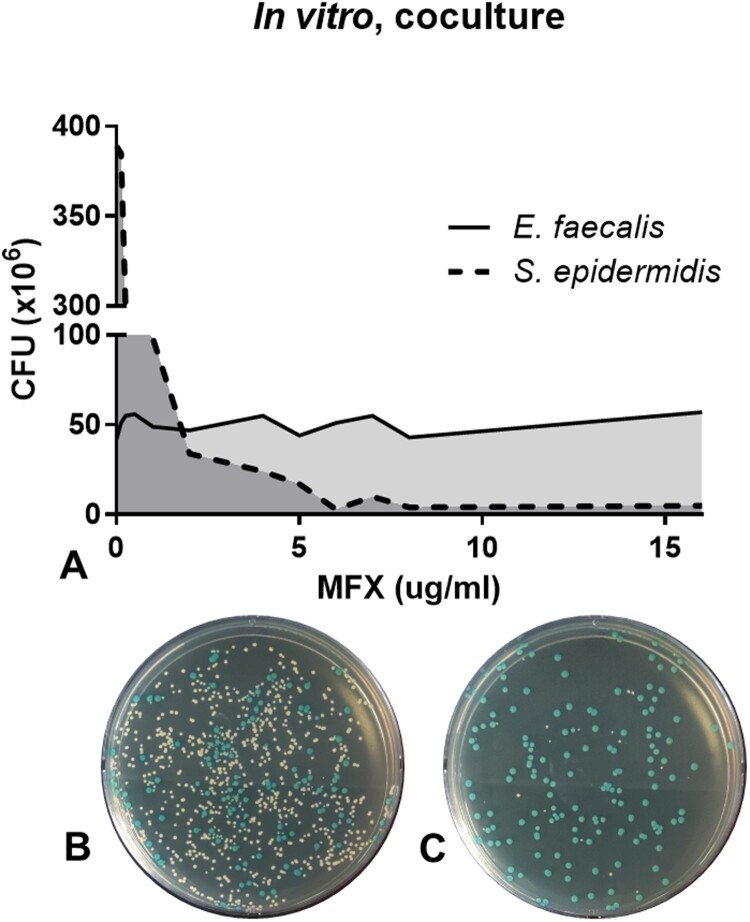
Survival assay of *S. epidermidis* and *E. faecalis* in *in vitro* co-culture under various concentrations of moxifloxacin (A). Representative bacterial culture plate images showing the distribution of the bacterial colonies on 0.25^ ^μg/mL moxifloxacin (B) and 16^ ^μg/mL moxifloxacin (C). Colour detection culture plates were used; *E. faecalis* are shown as blue colonies and *S. epidermidis* are shown as white colonies. CFU, colony forming unit; MFX, moxifloxacin.

*In vitro* co-culture of *E. faecalis* and *S. epidermidis* was performed to understand the growth effect between two bacterial species and the effect of antibiotics on both species. Even though the equal number of bacterial cells of two species were co-cultured without antibiotics, *S. epidermidis* outgrew *E. faecalis* with approximately 8:1 ratio (Figure 1A, data on 0 µg/mL moxifloxacin). When moxifloxacin was added to the co-culture, the colony numbers of *S. epidermidis* started to decrease from 0.25 µg/mL moxifloxacin and reached less than 10 CFU at 6.0 µg/mL moxifloxacin (Figure 1, Supplementary Table 2). On the contrary, the colony number *E. faecalis* was not changed even at 16.0 µg/mL moxifloxacin treatment, which was 64 times higher than *E. faecalis* MIC of moxifloxacin. These data show that the administration of moxifloxacin has an effect on the selective survival of *E. faecalis* in the *in vitro* co-culture setting at higher moxifloxacin concentrations.

### Survival assay in *in vivo* co-culture model

**Figure 2.**
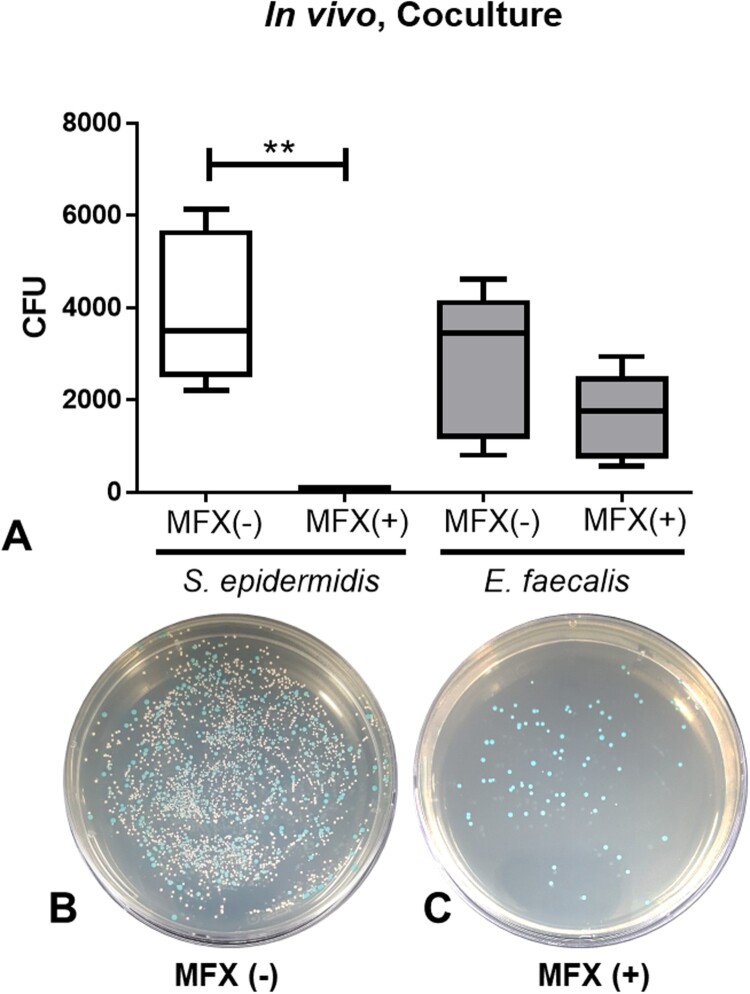
Survival assay of *S. epidermidis* and *E. faecalis* in *in vivo* co-culture after administration of 0.5% moxifloxacin (A). Representative bacterial culture plate images showing the distribution of the bacterial colonies on the absence (B) and presence (C) of moxifloxacin topical drops. Colour detection culture plates were used. *E. faecalis* are shown as blue colonies and *S. epidermidis* are shown as white colonies. **, *p* < 0.005, CFU, colony forming unit; MFX, moxifloxacin; MFX (+), moxifloxacin-treated; MFX (−), moxifloxacin-untreated.

Here we further investigated the selective survival of *E. faecalis* under moxifloxacin exposure in the *in vivo* co-culture model. The number of colonies of *S. epidermidis* and *E. faecalis* in the antibiotics untreated co-culture (CO) group were 369 and 281.9 CFU, respectively, and that in moxifloxacin treated co-culture (MC) group were 62 and 164.7 CFU, respectively (Figure 2). The number of colonies of *S. epidermidis* in the CM group was significantly lower than that of the CO group (*p* = 0.0008), but the number of colonies of *E. faecalis* did not show a significant difference between the two groups (*p* = 0.1973). Administration of moxifloxacin drops (twice, 5 µL, 5 mg/mL) revealed antibiotic-selective survival of *E. faecalis* in comparison to *S. epidermidis in vivo*.

## Discussion

This study showed that *Enterococcus* spp. has been one of the major causative microorganism of PE. To find out the reason for the increase of *Enterococcus* spp., we investigated if there were changes in the normal conjunctival microbes and established *in vitro* and *in vivo* co-culture model of *E. faecalis* and *S. epidermidis*. There was no domination of *Enterococcus* spp. in conjunctival microbes and *E. faecalis* selectively survived under the pressure of fourth-generation fluoroquinolone in the survival assays. This selective survival under fluoroquinolone could induce the domination of *Enterococcus* spp. on conjunctiva perioperatively, enabling *Enterococcus* spp. to enter the intraocular space and causing PE.

In the past, the incidence of *Enterococcus* spp. in PE was reported to be only about 2.2% in The Endophthalmitis Vitrectomy Study (EVS), showing much lower incidence than that of CNS (70.0%) [5]. However, in addition to South Korea, the incidence of *Enterococcus* spp. has been increasing in Sweden, Japan, and Taiwan [4,7,8,21]. A recent report in Taiwan showed that *Enterococcus* spp. was the most common isolate (38.1%) of PE, followed by *S. epidermidis* (28.6%) [8]. Kim et al. and Friling and Montan speculated that the intrinsic resistance of *Enterococcus* spp. to broad spectrum antibiotics used perioperatively might be associated with the emergence based on the antibiotic susceptibility results of microorganisms cultured from PE [4,10]. However, whether these approaches provide a valid explanation for the emergence of *Enterococcus* spp. as a major causative microorganism of PE has been uncertain for the following reasons. The clinical antibiotic susceptibility reports collected from many clinical laboratory departments of individual institutes were varied, often not including antibiotics that are commonly used in eye clinic such as fluoroquinolone to be tested on the *Enterococcus* spp. isolates. Moreover, the CLSI standard for recent fluoroquinolone drugs for *Enterococcus* spp. has not been defined. Therefore, in this study, we tried to test the hypothesis that the selection pressure of perioperative antibiotics is the cause of the rising *E. faecalis* as dominant causative microorganisms of PE by investigating the effect of fourth generation fluoroquinolone in *in vitro* and *in vivo* co-culture model of *E. faecalis* and *S. epidermidis*.

Because the source of bacteria causing PE mostly comes from the patient’s own conjunctival microbes [20,22], we investigated microbes distribution on conjunctiva before cataract operation or intravitreal injection. The proportions of *S. epidermidis* and *E. faecalis* in conjunctival microbes were 33.0% and 4.7% in this study. In other studies, CNS was the most common isolate, with a proportion of 28.6-72.4%, and *Enterococcus* spp. was rarely isolated from normal conjunctiva, while several studies isolated *Enterococcus* spp. from 2.5% to 5.2% in their cases [23–27]. We observed similar pattern of microorganisms population isolated from normal conjunctiva to the previous studies.

Next, we examined fluoroquinolones susceptibility of *E. faecalis* and *S. epidermidis* isolated from the conjunctiva. The MIC of *E. faecalis* standard strain on fluoroquinolones was two to four times higher than that of *S. epidermidis* standard strain in the different generations of fluoroquinolones. In clinical isolates, the proportion of resistant *E. faecalis* to fluoroquinolones was much higher than that of *S. epidermidis*.

The difference of antibiotic susceptibility between *E. faecalis* and *S. epidermidis* to fluoroquinolones could be a clue implying the selective survival of *E. faecalis* under broad spectrum antibiotics. *In vitro* co-culture of *E. faecalis* and *S. epidermidis* data showed that *E. faecalis* can survive in response to antibiotics (from 2 to 16 µg/mL) more than *S. epidermidis*. Surprisingly, under an extremely high concentration of moxifloxacin (16 µg/mL), the colony number of *E. faecalis* was maintained in contrast to that of *S. epidermidis*. Without moxifloxacin, *S. epidermidis* is about eight times more populated than *E. faecalis* in *in vitro* co-culture model. However, *E. faecalis* started to populate more than *S. epidermidis* at the moxifloxacin concentration of 1–2 µg/mL. Considering that the concentration of moxifloxacin on the market is 0.5%, which corresponds to 5 × 10^3 ^µg/mL, one drop of moxifloxacin is about 0.05 mL containing 250 µg moxifloxacin. This phenomenon was also observed in the *in vivo* animal experiment. When the colony number of *E. faecalis* and *S. epidermidis* in conjunctiva were compared after moxifloxacin drops, the colony number of *E. faecalis* was significantly greater than that of *S. epidermidis*. Our data suggest the selective survival of *E. faecalis* in the *in vivo* co-culture rabbit model under the pressure of moxifloxacin. According to an existing animal study, when a single drop of 0.3% moxifloxacin was topically administrated in rabbit eyes, the concentration in the tear film dropped to less than 10 µg/mL in a short time but remained above 1.0^ ^μg/mL upto the six hour after the administration [28]. These conditions might be sufficient for *E. faecalis* to selectively survive on conjunctiva over other Gram positive microorganisms, including *S. epidermidis*.

In conclusion, we propose that the increase in *Enterococcus* spp. as a cause of PE may result from selective pressure of perioperative broad spectrum antibiotics such as fourth-generataion fluoroquinolones. Currently, the fourth-generation fluoroquinolone is the most commonly used antibiotic for the prevention of infection after cataract operation globally including Korea. These changes are very likely to be observed worldwide. Of concern, the increase of *Enterococcus spp.* isolate in PE has poor impact on the visual outcome of endophthalmitis [3,4,11,12,29]. To address this challenge, various efforts will be needed in the near future.

## Supplementary Material

Supplemental Material
