# Supplementary material for: Emerging Enterococcus isolates in postoperative endophthalmitis by selection pressure of fluoroquinolones: an 11-year multicenter and experimental study
Source: Emerg Microbes Infect. 2020 Aug 28;9(1):1892–9. doi: 10.1080/22221751.2020.1810134 (PMC7473211; doi:10.1080/22221751.2020.1810134)
Supplement: Supplemental Material [file TEMI_A_1810134_SM6213.docx]

Supplementary method.

1. Cultue and identification method of clinical samples

For clinical samples, direct smears by Gram, gomorimethenamine silver, periodic acid Schiff staining and culture on blood agar, chocolate agar, thioglycolate broth were performed. Matrix-Assisted Laser Desorption Ionization-Time of Flight Mass Spectrometry (MALDI-TOF MS, Bruker Daltonics GmbH, Germany) was performed to identify the microorganisms using an identification card (VITEK 2 ID Card, bioMérieux, USA). Antimicrobial susceptibility was tested using an automated system (Phoenix, Becton Dickinson Diagnostic Systems, Sparks, MD, USA; VITEK 2, bioMérieux, Durham, NC, USA; or MicroScan Walk-Away, Dade Behring, Inc., West Sacramento, CA, USA). The CLSI standards were used for interpretation and quality control for each corresponding year.

1. Bacterial strains and growth condition

The standard strains of *E. faecalis* ATCC29212 and *S. epidermidis* KCTC1917 (equal to ATCC12228) were purchased from American Type Culture Collection and Korean Collection of Type Cultures, respectively. Both strains were cultured in tryptic soy broth (TSB) or on the tryptic soy agar (TSA) at 37°C. The standard strains were used for *in vitro* and *in vivo* co-culture model.

1. Broth micro-dilution antibiotic susceptibility test

A serial broth micro-dilution method recommended by CLSI was performed to determine minimal inhibition concentration (MIC) of fluoroquinolone antibiotics. Bacteria cells were streaked and cultured on TSA plate for 24 hours to generate single colonies. The single colonies were repeatedly resuspended in normal saline in order to match the turbidity of bacterial suspension to 0.5 McFarland standard at 600 nm spectrophotometry (Mecasys Co. Ltd, Korea). The suspension was further diluted using cation adjust Muller Hinton broth (CAMHB) to have a bacterial solution of 5 x 10^6^ CFU/mL. Next, ten microliter of the bacterial solution was added to 90 µl of CAMHB containing two-fold dilution of antibiotics in the each well of 96 well plate. After incubation at 37°C for 24 hours, the growth of bacteria was examined by observing turbidity of each well and further visualized by adding MTT (3-(4,5-dimethylthiazol-2yl)-2,5-diphenyl tetrazolium bromide, Sigma, USA) with a final concentration of 500 µg/mL, which exhibit the blue color indicating the growth of bacteria. The lowest concentration of antibiotics inhibiting the growth of bacteria was determined as MIC.

Supplementary table 1. Minimal inhibitory concentration of standard strains was used for the cut-off value for clinically isolated *Staphylococcus epidermidis* and *Enterococcus faecalis*.

| Microorganism | | Ciprofloxacin (μg/mL) | Levofloxacin (μg/mL) | Moxifloxacin (μg/mL) |
| --- | --- | --- | --- | --- |
| *S. epidermidis* | KTCT1917 | 0.25 | 0.25 | 0.125 |
| *E. faecalis* | ATCC 29212 | 1 | 1 | 0.25 |

Supplementary table 2. Survival assay of *S. epidermidis* and *E. faecalis* in *in vitro* co-culture under various concentrations of moxifloxacin. The colony number of *S. epidermidis* and *E. faecalis* was counted in three culture plates under various concentrations of moxifloxacin.

|  | *S. epidermidis* | | | | *E. faecalis* | | | |
| --- | --- | --- | --- | --- | --- | --- | --- | --- |
| Dilution factor | 10^5^ | | 10^6^ | | 10^5^ | | 10^6^ | |
| MFX conc. (μg/mL) | Average | SD | Average | SD | Average | SD | Average | SD |
| Control | TNTC |  | 389 | 23.7 | TNTC |  | 42 | 6.5 |
| 0.125 | TNTC |  | 385 | 2.4 | TNTC |  | 51 | 3.7 |
| 0.25 | TNTC |  | 284 | 24.5 | TNTC |  | 55 | 3.7 |
| 0.5 | TNTC |  | 209 | 10.6 | TNTC |  | 56 | 9.4 |
| 1 | TNTC |  | 98 | 5.7 | TNTC |  | 49 | 1.6 |
| 2 | 127 | 7.8 | 34 | 7.3 | 431 | 6.5 | 47 | 4.1 |
| 4 | 64 | 25.7 | 24 | 3.7 | 441 | 43.7 | 55 | 2.9 |
| 5 | 26 | 1.2 | 17 | 1.6 | 454 | 37.6 | 44 | 6.1 |
| 6 | 18 | 0.4 | 3 | 2.0 | 476 | 40.4 | 51 | 6.5 |
| 7 | 39 | 9.8 | 10 | 3.7 | 458 | 20.8 | 55 | 16.7 |
| 8 | 13 | 0.8 | 4 | 0.4 | 466 | 55.9 | 43 | 3.3 |
| 16 | 11 | 1.6 | 5 | 0.0 | 406 | 64.9 | 57 | 2.9 |

TN, Too much to count; SD, standard deviation; MFX, moxifloxacin; conc., concentration
